# Effects of Episodic Future Thinking and Self-Projection on Children’s Prospective Memory Performance

**DOI:** 10.1371/journal.pone.0158366

**Published:** 2016-06-29

**Authors:** Anett Kretschmer-Trendowicz, Judith A. Ellis, Mareike Altgassen

**Affiliations:** 1 Department of Psychology, Technische Universitaet Dresden, Dresden, Germany; 2 School of Psychology & Clinical Language Sciences, University of Reading, Reading, United Kingdom; 3 Donders Institute for Brain, Cognition and Behaviour, Radboud University, Nijmegen, The Netherlands; University College London, UNITED KINGDOM

## Abstract

The present study is the first to investigate the benefits of episodic future thinking (EFT) at encoding on prospective memory (PM) in preschool (age: *M* = 66.34 months, *SD* = 3.28) and primary school children (age: *M* = 88.36 months, *SD* = 3.12). A second aim was to examine if self-projection influences the possible effects of EFT instructions. PM was assessed using a standard PM paradigm in children with a picture-naming task as the ongoing activity in which the PM task was embedded. Further, two first- and two second-order ToM tasks were administered as indicator of children’s self-projection abilities. Forty-one preschoolers and 39 school-aged children were recruited. Half of the participants in each age group were instructed to use EFT as a strategy to encode the PM task, while the others received standard PM instructions. Results revealed a significant age effect, with school-aged children significantly outperforming preschoolers and a significant effect of encoding condition with overall better performance when receiving EFT instructions compared to the standard encoding condition. Even though the interaction between age group and encoding condition was not significant, planned comparisons revealed first evidence that compared to the younger age group, older children’s PM benefitted more from EFT instructions during intention encoding. Moreover, results showed that although self-projection had a significant impact on PM performance, it did not influence the effects of EFT instructions. Overall, results indicate that children can use EFT encoding strategies to improve their PM performance once EFT abilities are sufficiently developed. Further, they provide first evidence that in addition to executive functions, which have already been shown to influence the development of PM across childhood, self-projection seems to be another key mechanism underlying this development.

## Introduction

Remembering to perform intentions after a delay—prospective memory (PM)—is an important daily challenge and a precursor for independent living. PM development across the lifespan can be modeled using an inverted U-shaped function (e.g. [1,2]). While a large number of studies have focused on PM in older adults (see [3,4] for meta-analyses), PM development at the other end of the lifespan has long been neglected (see [5] for a review addressing this issue). During the last few years, however, PM in childhood and adolescence has become an important focus of research. Studies point to a developmental increase in performance in both time-based (i.e. remembering future intentions at a certain time-point, e.g. [6,7]) and event-based (i.e. remembering to perform future intentions when a specific PM cue is presented, e.g. [8–10]) PM tasks. This improvement has been related to continuously developing executive functions (see [11] for a review) as well as the development of prefrontal brain structures that represent important neural correlates of both executive functions and PM (for PM see e.g. [12,13], for executive functions see e.g. [14]). Similar to PM, executive functions develop from childhood to adolescence (e.g. [15,16]).

PM is a complex process consisting of four phases with different executive functions playing a key role [17,18]. The first phase includes the formation of an intention that needs to be executed in the future, where *encoding* as well as *planning* when and how to perform the PM task is crucial. This is followed by intention retention when the individual is engaged in other ongoing activities and *retrospective memory* is needed to retain the PM task content. When the PM cue appears in a subsequent ongoing activity, the intended action has to be recalled, initiated and executed. To initiate the intended action, the environment often needs to be *monitored* for the occurrence of the PM cue and the ongoing activity needs to be *inhibited* to *switch* to the PM task at the appropriate moment (see [18] for further details).

A growing body of research is systematically exploring the role of executive functions in the development of children’s PM. Ford, Driscoll, [19] found that inhibition significantly contributed to event-based PM in 4- to 6-year-old children. In contrast, Mahy and Moses [20] reported that working memory but not inhibition was a significant predictor of event-based PM in a similar age group. Mackinlay, Kliegel [21] investigated time-based PM in 7- to 12-year-olds and showed that both planning and switching contributed significantly to PM. Overall, results support a potentially crucial role for executive functions in PM task success.

According to the influential multiprocess framework [22], the extent to which PM tasks are executed strategically (strong reliance on cognitive control processes) or relatively automatically depends on task characteristics such as PM cue salience (e.g. [9]), PM task importance (e.g. [23]) or ongoing task difficulty (e.g. [24]). Specifically, McDaniel and Einstein [22] propose that cognitive control and thus strategic processing is more strongly involved in PM when cues have low perceptual salience (cf. ongoing task items, thus requiring more monitoring for detection), the importance of the PM task is emphasized (i.e., the individual employs additional monitoring to ensure better performance) and when the ongoing task is less demanding (releasing cognitive processes for PM task completion). Another important PM cue characteristic that is proposed to impact on the involvement of strategic processes in PM task performance is the focality of the PM cue. PM cues that are focal to the ongoing task require the same information to be processed as ongoing task items and are thus assumed to be detected relatively automatically [22]. In contrast, non-focal PM cues require more cognitive control processes to successfully perform the PM task (i.e., monitoring for a cue that is not central for ongoing task completion, inhibiting ongoing task performance at the appropriate moment, and switching from the ongoing to the PM task; see also [25]). In addition to the above task characteristics, planning during the intention formation phase may impact on the need for cognitive control and strategic processes. In support of this assertion, relationships between plan performance and PM have been observed for different populations (e.g., individuals with autism [26], older adults [27]). Similarly, with regards to encoding strategies, studies applying implementation intentions (i.e., specific plans that link future situations to predefined responses in the structure of an if-then statement, [28,29]) during intention encoding reported improved PM performance and reduced PM age differences (e.g. [30,31]). Several studies have augmented the verbal if-then statement with an imagery component [30] and asked participants to imagine themselves performing the PM task later on (see e.g. [32] and experiment 1 of [33] for further evidence). Overall, studies found improved PM performance when implementation intentions and imagery were instructed during intention encoding. McFarland and Glisky [34] directly tested whether adding an imagery component to implementation intentions extended the effects of ‘if-then’ plans. Specifically, three encoding conditions were tested, namely implementation intentions only, imagery only and implementation intentions combined with imagery. The authors found that *all* three encoding conditions similarly improved PM in a sample of young adults. Thus, not only implementation intentions (or a combination of them with imagery), but also imagery alone increased PM performance. Recently, Schacter, Addis [35] have argued that future event simulation (resp., imagery) may automatically occur (at least to some extent) while the prospective intention is encoded. Thus, the ability to simulate future events may be especially important for planning future activities. Episodic future thinking (EFT) is defined as the projection of oneself into the future to mentally pre-experience future situations. It encompasses the multimodal re-construction of past experiences and projects them into the future [36,37]. Both PM and EFT describe future-oriented concepts and consistent with this assertion EFT has been shown to be related to the intention formation phase of PM [25,38]. Moreover, Schacter, Addis [35] observed an overlap in brain activation, particularly rostral prefrontal cortex, during the performance of EFT and PM tasks, providing evidence for a common neural basis for PM and EFT (for evidence of Brodmann area 10 involvement in PM see [13], for further evidence on Brodmann area 10 involvement in future event simulation see [39]). It is somewhat surprising, therefore, that few studies have explicitly targeted relations between EFT and PM or more specifically, whether EFT during intention encoding may improve PM performance. Neroni, Gamboz [38] investigated PM in a student population and tested whether asking participants to mentally simulate PM task execution (starting with arriving at the university and ending with leaving the lab) enhanced PM on the following day. They found beneficial effects of EFT instructions (i.e., imagining later PM task execution) on performance when the PM task matched the mentally simulated task from the previous day. Importantly, PM did not improve when participants worked on a control task or mentally simulated an unrelated task (for further evidence on the role of contextual information included in the imagery see [40]). Recently, Altgassen, Rendell [25] reported beneficial effects of EFT instructions on PM and plan adherence in younger and older adults in a PM task with high planning demands. Overall, the beneficial effects of implementation intentions and EFT have been suggested to promote deeper encoding of the prospective intention as well as stronger cue-action associations as a result of these encoding instructions. This, in turn may result in relatively automatic retrieval of the prospective intention (e.g. [41]) when engaging in the future situation. This reduction in cognitive control demands should especially benefit the PM performance of individuals with still developing (children) or already decreasing (older adults) cognitive control capacities. For older adults those beneficial effects have already been demonstrated, while surprisingly no study has addressed this issue in children.

Developmental studies indicate that EFT starts to develop between 3 and 4 years of age [42] and increases across childhood and adolescence ([43], see [44] for a review). Even though relations between EFT and PM have already been found in adult samples, research in younger age groups is still scarce and only two developmental studies have focused on preschool and school-aged children. Atance and Jackson [45] examined relations between PM and EFT in 3- to 5-year-olds and found a significant correlation that, after partialling out age, was no longer significant. Similarly, Nigro, Brandimonte [46] investigated the relation between PM and EFT in 4- to 7-year-olds. To measure EFT, they applied a task developed by Atance and O’Neill [42,47] asking children to pack a bag for a near-future trip. The PM task was to remind the experimenter to return a mobile phone to a colleague. Results showed a significant relation between PM and EFT in 7-year-old children, but not in younger age groups. Furthermore, age and EFT were significant predictors of PM. However, both studies applied correlational designs and did not systematically manipulate the extent to which participants engaged in EFT during intention formation. This omission is surprising, given that explicitly instructing EFT as an encoding strategy might help children to succeed in PM tasks, as they may not automatically make use of EFT while encoding the future intention. To date, studies on the effects of EFT encoding on PM performance have mostly addressed adult populations and only one recent study investigated effects of EFT encoding on adolescents’ PM [48]. Specifically, this study tested effects of future thinking on adolescents’ and young adults’ PM and contrasted this encoding condition with a repeated-encoding (i.e., repeatedly presenting the ongoing task and the PM cues for two minutes) and a standard encoding condition. The authors reported adolescents to benefit similarly from future thinking *and* the repeated-encoding condition, while PM performance between these two encoding conditions did not differ. Thus, EFT seems to represent a strategy that can improve adolescents’ PM. The effectiveness of this strategy on children’s PM, however, remains unknown given the lack of empirical evidence and the still ongoing development of EFT abilities across childhood [43]. The present study investigated the effects of EFT encoding on PM performance in preschool and school-aged children.

Conceptually, both PM and EFT are future-oriented cognitive concepts and have recently been related to Theory of Mind (ToM). ToM is defined as the ability of mentally taking perspectives of other people to understand their beliefs that starts to develop in 4- to 6-year-old children [49]. Interestingly, it has been proposed that *self-projection* as the ability to shift from the current perspective to an alternative perspective [50] underlies these relations [19] and may also enable individuals to explore different temporal perspectives and to imagine and pre-experience future situations (EFT, [19,50]) or to remember the past (episodic memory, [35,50]). Thus, measures of ToM serve as indicators of self-projection abilities. Only recently, research has begun to explore the role of self-projection on the performance of delayed intentions in children (19) and only three studies have addressed possible relations between PM and ToM. In two experiments, Ford, Driscoll [19] found that ToM significantly contributed to PM in 4- to 6-year-old children. Similarly, Williams, Boucher [51] examined effects of ToM on PM in children with autism and reported relations between time-based PM impairments and difficulties with ToM. In a recent study with adolescents, Altgassen, Vetter [52] found ToM to predict event-based PM, in addition to adolescents’ switching abilities. Taken together, studies suggest that ToM (and thus possibly self-projection) contributes to PM. In a recent paper, Hanson, Atance [53] explored relations between EFT, executive functions and ToM in preschool children. Although, the authors found significant correlations between all three concepts, arguing for behavioral commonalities, these relations were no longer significant after controlling for age and language. Importantly, this study focused on preschoolers and did not target possible relations in school-aged children. Hanson, Atance [53] suggested that relations between EFT and ToM may only emerge in older children. However, to the best of our knowledge nothing is known about the interplay between mentally engaging in future situations, self-projection and prospective remembering. Therefore, in addition to testing whether EFT instructions improve children’s PM performance, the second aim of the present study was to investigate whether these effects were influenced by children’s self-projection abilities (as measured by ToM tasks). This issue was addressed in samples of 5- and 7-year-olds using a standard PM paradigm with varying encoding conditions and well-established measures of ToM performance to test children’s self-projection abilities.

Regarding the effects of EFT encoding on children’s PM two opposing hypotheses were considered: The first is that younger children should benefit more from EFT instructions given that their executive functions are less developed than those of older children. Therefore, they should benefit more strongly from the supposedly deeper encoding of the intention and the decreased need for strategic monitoring during the delayed performance interval following EFT instructions. The second possibility that has emerged from previous literature is that older children might benefit more from EFT because their abilities to mentally simulate future events are further developed than that of their younger counterparts [43], which may enable them to make more use of EFT to improve their PM performance. Finally, we explored whether possible effects of EFT instructions on PM performance were driven by the development of self-projection as measured by ToM.

## Method

### Participants

Eighty-two children participated in this study. One preschool and one primary school child were excluded due to failing to understand PM instructions, resulting in a final sample of 80 participants. The preschool group consisted of 41 children (age in months: *M* = 66.34, *SD* = 3.28; age range: 60 to 71 months), and the primary school group comprised 39 children (age in months: *M* = 88.36, *SD* = 3.12; age range: 85 to 95 months). Children were recruited through kindergartens and local primary schools. All had German as their first language, were in good health and had no psychiatric or developmental disorders as measured by a socio-demographic questionnaire completed by their parents. There were no significant differences between the two age groups in either gender distribution (preschool group: 24 female, 17 male; primary school group: 17 female, 22 male; *Χ*^*2*^(1, *N* = 80) = 1.79, *p* = .18) or an age-normed measure of verbal abilities (preschool group: *M* = 11.76, *SD* = 2.18; primary school group: *M* = 11.26, *SD* = 1.77; *F*(1,78) = 1.26, *p* = .27, η^2^_p_ = .02).

The study was approved by the ethics committee of the medical faculty of the University of Dresden (Ethikkommission der Medizinischen Fakultät, Technische Universität Dresden). Children were allowed to participate if their parents gave written informed consent.

### Materials

#### PM assessment

A version of Kvavilashvili, Messer’s [10] PM paradigm was employed to assess PM. This paradigm comprises a card-naming ongoing task and a PM task, which consisted of remembering to hide certain pictures. Moreover, to maintain children’s motivation Kvavilashvili et al. used the toy mole “Morris” who needed children’s help. Following this approach, we also introduced a card-naming task as ongoing activity with embedded PM task as well as the toy mole that needed help to get along in the world. For the ongoing task children had to name objects presented as black-and-white line drawings on picture cards taken from the standardized picture system of Snodgrass and Vanderwart [54]. Children had approximately 3 seconds to name the objects. If no answer was made within this time window, the next picture was presented. Following Sheppard, Kretschmer’s [55] approach, baseline ongoing task performance was assessed by asking children to name 10 objects. Moreover, the number of ongoing task items presented during the PM block (ongoing and PM tasks) was adapted for the two age groups. Preschoolers were required to name 60 cards, whereas school children had to name 80 cards. The PM task was to say the German word for juice (“Saft”) and turn the card over whenever a fruit or a vegetable appeared on the card. Each stack included three PM cues, giving a maximum of 12 PM hits in both age groups. To ensure all children knew the fruits and vegetables used in the task and were able to include them in the subsequent imagery task, they were asked to list the fruits and vegetables that they knew. Dependent variables were proportions of correct PM and ongoing task hits.

The PM task was introduced in two different conditions. Half of the participants in each age group had to mentally simulate the execution of the PM task while encoding the prospective intention (EFT condition), while the other half received standard PM instructions (standard condition). In the *EFT condition* children were instructed to imagine traveling through time. To illustrate this, a remote control was introduced and children told they should imagine that it is possible to go back and forward in time by clicking two of its buttons. Before mentally simulating the PM task, children were provided with practice on EFT by imagining giving a drawing to their mother on arrival home. Children were guided through this exercise with questions (e.g., “How does your mother look like?”, “What are you going to say to your mother?”) and asked to close their eyes and imagine the event in as much detail as possible. After this practice, they were required to imagine the future situation when performing the PM task later. Again they were asked to close their eyes and imagine as many details as possible while the experimenter described the future situation of playing the card game. Children had to imagine the experimenter showing the cards and themselves naming them and to imagine the situation when a fruit or a vegetable appeared on the cards and what they would do. To provide a vivid image, children had to imagine the environment, the experimenter and what they might hear, feel and smell while playing the card game. They were told it was important to imagine the game as if they were playing it. In the *standard condition*, children received standard PM instructions to remember to say the word ‘juice’ whenever the picture of a fruit or vegetable was presented.

#### ToM assessment

Two first- and two second-order ToM tasks were selected as indicators for individuals’ self-projection abilities. First-order ToM tasks require individuals to reason about other individuals’ thoughts, whereas second-order ToM tasks address the ability to reason about what one person is thinking about another person’s thoughts (e.g. [56]). First-order ToM was investigated with the Sally-Ann [57] and Smarties task [58]. Second-order ToM was assessed with adapted versions of Perner and Wimmer’s [59] ice-cream-truck task and the bake-sale story (both taken from [60]). All ToM tasks used puppets, materials described in that task and maps to introduce protagonists to the child and show how they were moving around during the story. During all ToM stories control questions are asked to ensure correct understanding. The Sally-Ann task is a prominent first-order ToM task, where Sally puts a ball in her basket and leaves the room. Ann takes the ball out of Sally’s basket and puts it into her box while Sally is outside the room. The following questions are asked: Who is Sally, who is Ann? (control question 1), Where is Sally going to look after her ball when she returns? (first-order ToM question), Where was the ball at the beginning? (control question 2) and Where is the ball at the end? (control question 3). The Smarties task follows a similar approach with a different story (see [58]). Also here, the maximum number of first-order ToM hits is one and three control questions are asked. In both second-order ToM tasks children are told stories about two protagonists (e.g., ice-cream-truck task: Marie and Johann; names adapted from the original version). At the beginning the protagonists have the same belief (e.g., Marie and Johann are in the park, where ice cream is sold. The ice-cream man says that he will be in the park in the afternoon. Marie is leaving the park.). Then, the belief of one protagonist changes without the other protagonist knowing (The ice-cream man leaves the park with Johann but not Marie seeing this. The ice-cream man tells Johann that he will go to a street close to the church). Control question 1) follows: Has Marie heard how the ice-cream man told this Johann? Subsequently, the other protagonist’s belief changes without the first one knowing (On the way to the church Marie meets the ice-cream man and he tells her that he is on the way to the church to sell ice cream). Children are then asked control question 2) Has Johann heard how the ice-cream man told this Marie? Hence, the first protagonist has a first-order belief that is different from the initial belief and from reality (Johann thinks that Marie is on her way to the park to buy ice cream.). The other protagonist knows the real situation, which differs from her second-order belief about the first protagonist (Marie knows that ice cream is sold at the church while Johann thinks that Marie thinks that the ice-cream man is in the park.). Later on Johann wants to visit Marie at home. Her mother tells him that Marie is on her way to buy ice cream. The following second order ToM questions are asked: 1) Where does Johann think Marie went to buy ice cream? 2) Why does Johann think that? Finally, two more control questions are asked: 3) Where did Marie go to buy ice cream? 4) Where was the ice-cream man at the beginning? Thus, a maximum of two second- order ToM points can be achieved, the maximum number of points for answering the control questions correctly is four. The bake-sales story task is similar with different protagonists and story (see [60]). Importantly, this task includes questions addressing first- as well as second-order ToM, resulting in a maximum of two first- and two second-order ToM hits. Five control questions are asked while reading the story. Taken together, the overall number of points, individuals could achieve for first-order ToM was four as was it for second-order ToM. For the present study, a combined score for ToM performance consisting of a maximum of four first- and four second-order ToM hits was calculated; proportions of ToM hits (i.e., achieved number of ToM hits / maximum number of ToM hits (8)) were used for the analyses.

#### Assessment of children’s verbal abilities

To assess participants’ verbal abilities the German version of the Wechsler Preschool and Primary Scale of Intelligence-Third Edition (WPPSI-III, [61]) was used for preschool children and the Wechsler Intelligence Scale for Children-Fourth Edition (WISC-IV, [62]), for primary school children, respectively. Within both tests, children have to explain words of increasing difficulty, which are read by the experimenter. For each word children receive zero, one or two points. The test is completed when all words were explained or when children give six answers scored with 0 points in the WISC-IV or five 0-point answers in the WPPSI-III. The maximum score of the WISC-IV is 68 points and for the WPPSI-III a maximum of 28 points can be received. For the analyses of the present study, raw scores were transferred to age-normed scores.

### Procedure

**Fig 1:**
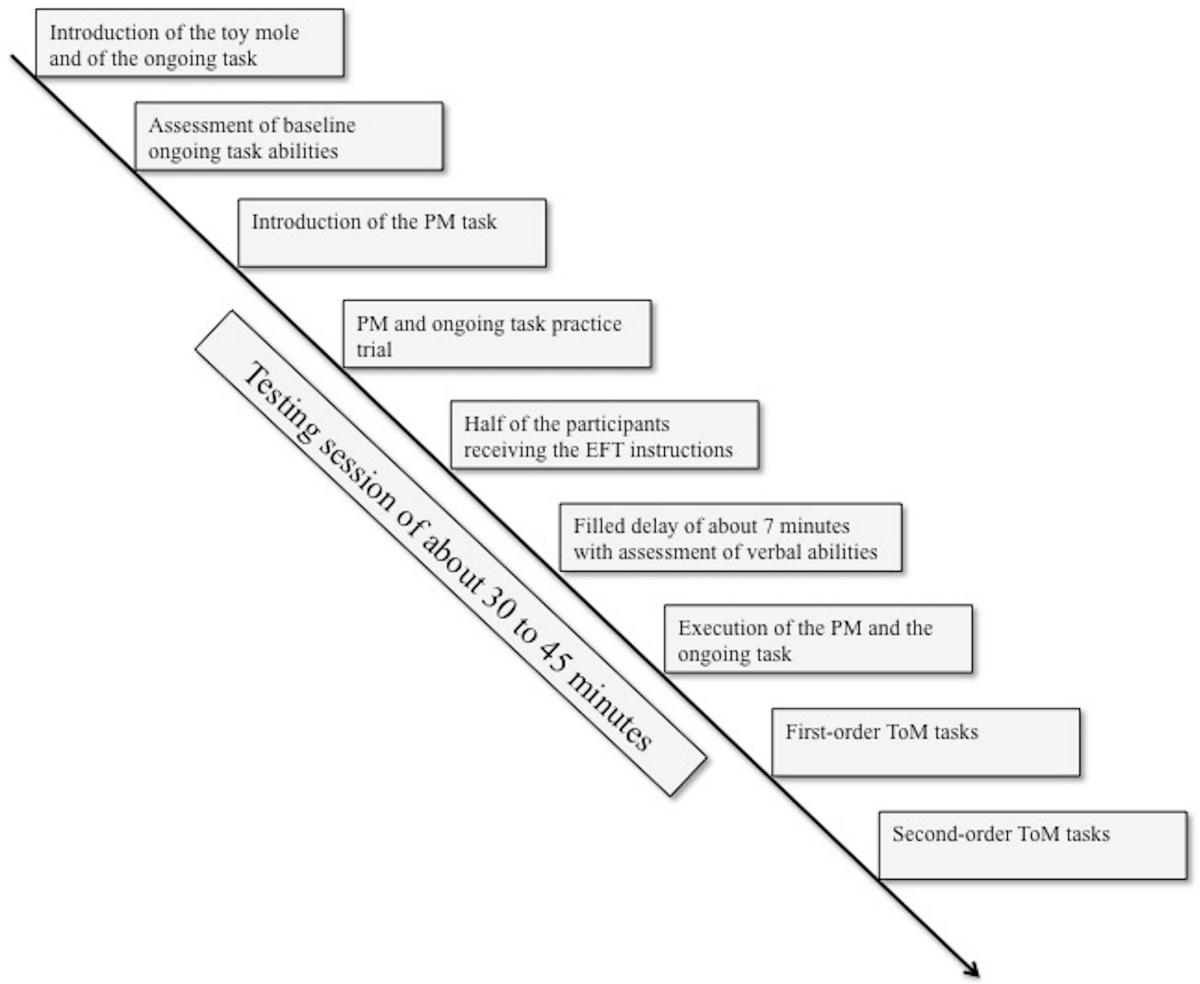
Task order within one testing session.

Fig 1 displays the order of tasks within one testing session.

After finishing the tasks, children were thanked for their participation and received a small toy.

## Results

Table 1 provides an overview of all descriptive measures of the present study, including age, gender, verbal abilities, PM, ongoing task and ToM performance.

**Table 1:** Descriptive measures of all included variables.

	Preschool children		School-aged children	
	EFT encoding	Standard encoding	EFT encoding	Standard encoding
	*N* = 21	*N* = 20	*N* = 20	*N* = 19
	*M(SD)*	*M(SD)*	*M(SD)*	*M(SD)*
Age	67.05 (3.35)	65.60 (3.12)	88.45 (3.47)	88.26 (2.79)
Gender	13 F, 8 M	11 F, 9 M	10 F, 10 M	7 F, 12 M
Verbal abilities	11.86 (2.48)	11.65 (1.87)	11.60 (1.60)	10.89 (1.91)
PM hits	0.50 (0.38)	0.33 (0.41)	0.79 (0.30)	0.41 (0.37)
Baseline ongoing task hits	0.93 (0.09)	0.93 (0.10)	0.99 (0.05)	0.96 (0.07)
Ongoing task hits in the PM task block	0.68 (0.05)	0.67 (0.06)	0.80 (0.03)	0.78 (0.07)
First-order ToM hits	0.54 (0.34)	0.60 (0.27)	0.88 (0.21)	0.72 (0.30)
Second-order ToM hits	0.21 (0.30)	0.14 (0.17)	0.54 (0.42)	0.40 (0.38)
Overall ToM performance	0.38 (0.29)	0.37 (0.15)	0.71 (0.25)	0.56 (0.25)
First-order ToM control question hits	0.95 (0.09)	0.97 (0.07)	0.98 (0.05)	0.94 (0.11)
Second-order ToM control question hits	0.71 (0.21)	0.66 (0.16)	0.92 (0.11)	0.88 (0.16)

*Note*. F = female, M = male. Age is displayed in months. PM, baseline ongoing task and ongoing task hits as well as all ToM measures are displayed as proportions of correct responses.

### PM performance and effects of EFT and self-projection

**Fig 2:**
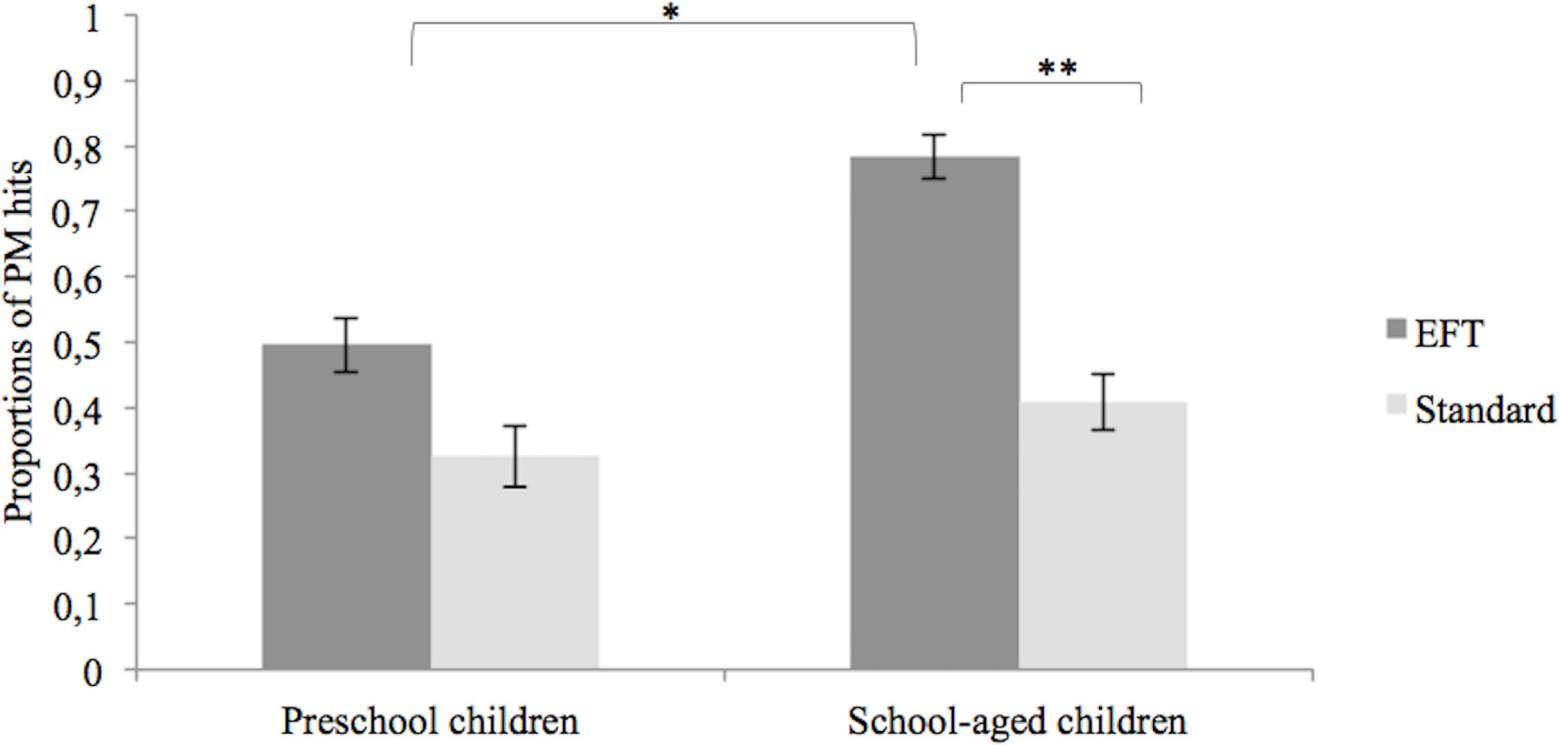
PM task performance for both age groups divided by encoding condition. Error bars depict standard errors of the mean.

A 2 (preschoolers, school-aged children) x 2 (EFT, standard) analysis of variance (ANOVA) was applied to test for age differences and the effects of EFT on PM. Results revealed main effects for age, *F*(1,76) = 5.14, *p* = .03, η^2^_p_ = .06, and encoding condition, *F*(1,76) = 11.12, *p* < .01, η^2^_p_ = .13. School-aged children outperformed preschoolers and overall children performed better when receiving the EFT compared to the standard PM instruction (see also Fig 2). The interaction between age group and encoding condition was not significant, *F*(1,76) = 1.59, *p* = .21, η^2^_p_ = .02. To explore *a priori* predictions, planned contrasts were calculated separately for encoding conditions and age groups. These revealed that school-aged children receiving the EFT instruction outperformed preschoolers in this condition, *F*(1,39) = 7.23, *p* = .01, η^2^_p_ = .16, whereas both groups performed similarly with standard PM instructions, *F*(1,37) = 0.44, *p* = .51, η^2^_p_ = .01. Moreover, the PM difference between EFT and standard condition was significant in the school-aged, *F*(1,37) = 12.38, *p* < .01, η^2^_p_ = .25, but not the preschool group, *F*(1,39) = 1.90, *p* = .18, η^2^_p_ = .05.

Importantly, both encoding groups were equated for age (preschool children: *F*(1,39) = 2.04, *p* = .16, η^2^_p_ = .05; school-aged children: *F*(1,37) = 0.03, *p* = .85, η^2^_p_ = .00), verbal ability (preschool children: *F*(1,39) = 0.09, *p* = .77, η^2^_p_ = .00; school-aged children: *F*(1,37) = 1.57, *p* = .22, η^2^_p_ = .04), gender (preschool children: *Χ*^*2*^(1) = 0.20, *p* = .65; school-aged children: *Χ*^*2*^(1) = 0.69, *p* = .41) and overall ToM performance (preschool children: *F*(1,39) = 0.01, *p* = .93, η^2^_p_ = .00; school-aged children: *F*(1,37) = 3.25, *p* = .08, η^2^_p_ = .08; for descriptive details see Table 1).

To explore the role of self-projection (overall ToM) on the effects of EFT on PM, in a first step, correlational analyses between PM and self-projection were conducted for each encoding group (EFT vs. standard) separately. Results revealed a significant relation between PM and self-projection in the EFT encoding group, *r*(39) = .46, *p* < .01, while this correlation was not significant in the standard encoding group, *r*(37) = .24, *p* = .15. To test whether the correlation between both concepts was influenced by age, the correlational analyses were repeated while partialling out age. Again, the relation between PM and self-projection was significant in the EFT group, *r*(36) = .34, *p* = .03, and not significant in the standard encoding group, *r*(34) = .19, *p* = .26. In a second step, the impact of self-projection on the effects of age and encoding condition on PM performance was tested using an exploratory ANCOVA with age group and encoding condition as independent variables, self-projection as covariate and PM performance as dependent measure. Results revealed a significant main effect of encoding condition, *F*(1,75) = 9.07, *p* < .01, η^2^_p_ = .11, with better PM performance in the EFT compared to the standard condition. The age group effect was no longer significant, *F*(1,75) = 0.80, *p* = .38, η^2^_p_ = .01. The effect of self-projection as a covariate was significant, *F*(1,75) = 5.75, *p* = .02, η^2^_p_ = .07. Finally, the interaction between age group and encoding condition was not significant, *F*(1,75) = 0.88, *p* = .35. η^2^_p_ = .01. Importantly, results also held, when excluding those participants from the analysis who failed to answer at least half of the ToM control questions correctly (effect of encoding condition: *F*(1,61) = 13.12, *p* < .01, η^2^_p_ = .18; effect of self-projection: *F*(1,61) = 6.14, *p* = .02, η^2^_p_ = .09; age group effect: *F*(1,61) = 1.86, *p* = .18, η^2^_p_ = .03).

### Ongoing task performance

A 2 (preschoolers, school-aged children) x 2 (EFT, standard) x 2 (baseline, PM task) mixed measures ANOVA, with proportion correctly named ongoing task pictures as dependent measure, was conducted to examine age differences in ongoing task performance. A significant main effect for age was revealed, *F*(1,76) = 47.41, *p* < .001, η^2^_p_ = .38, indicating that school-aged children correctly named significantly more pictures than preschoolers (see also Table 1). Both groups performed better in the baseline compared to the PM block, indicated by a significant main effect for ongoing task block, *F*(1,76) = 452.46, *p* < .001, η^2^_p_ = .86. The significant interaction between age group and ongoing task block showed that preschoolers deteriorated more strongly from the baseline to the PM condition than school-aged children (*F*(1,76) = 11.75, *p* < .01, η^2^_p_ = .13; see Table 1 for descriptive details). There was neither a main effect for encoding condition, *F*(1,76) = 1.95, *p* = .17, η^2^_p_ = .03, nor a significant interaction of this variable with age group, *F*(1,76) = 0.25, *p* = .62, η^2^_p_ = .00.

To test whether effects of encoding condition on PM performance still held when controlling for baseline ongoing task performance a 2 (preschoolers, school-aged children) x 2 (EFT, standard) ANCOVA with baseline ongoing task performance as covariate was conducted. Results revealed a significant main effect for encoding condition, *F*(1,75) = 10.19, *p* < .01, η^2^_p_ = .12, but neither a significant age effect, *F*(1,75) = 2.74, *p* = .10, η^2^_p_ = .04, nor a significant interaction between age and encoding condition, *F*(1,75) = 1.44, *p* = .23, η^2^_p_ = .02. The effect of baseline ongoing task performance appeared to be significant, *F*(1,75) = 4.34, *p* = .04, η^2^_p_ = .06.

## Discussion

This study is the first to investigate the effects of EFT on PM in children by experimentally manipulating encoding of the PM task. Moreover, we tested whether self-projection (indicated by ToM) had an impact on the effects of EFT instructions.

With regards to the effects of EFT instructions on PM performance, two opposing hypotheses were tested, namely 1) Preschool children benefit most from the deepened encoding and the strong cue-action link following EFT encoding, 2) School-aged children benefit more from the instruction of EFT, because their EFT abilities might be further developed.

Overall, results of the present study point to a PM improvement while transiting from preschool- to school-age, consistent with a number of studies investigating children’s PM (e.g. [9,10,63], see [11] for a review). Addressing the effect of EFT encoding on PM and thus our first research question, at a first glance the significant main effect for encoding condition indicates that both age groups benefited from EFT instructions. However, planned analyses revealed a significant PM improvement for 7-year-old but not 5-year-old children following EFT encoding instructions. Moreover, both age groups performed similarly under the standard encoding condition, whereas older children made more correct PM responses than younger children in the EFT condition. Thus, our results provide support for our second hypothesis, namely that older children benefit more from instructing EFT, given their better developed abilities to imagine future situations [43]. By experimentally manipulating EFT in a PM task, our study extends Nigro, Brandimonte’s [46] correlational results with children from a similar age range (4- to 7-year-olds). Consistent with Nigro, Brandimonte [46], the present study also observed beneficial effects of EFT instructions on PM in 7- but not 5-year-olds. Results of both studies suggest that future thinking skills are not sufficiently developed in younger children to significantly support PM. Older children and adolescents, whose future thinking abilities are known to be further developed [43] seem to be able to benefit more from EFT instructions, and consequently were able to improve their PM (see [48] for evidence on adolescents). Interestingly, Altgassen, Rendell [25] investigated the effects of EFT on PM in younger and older adults and showed that even though older adults’ abilities to imagine future situations are diminished (e.g. [64]), they can benefit from EFT instructions as much as younger adults. In early development this pattern seems to differ. Possibly, older adults compensate for declining future thinking abilities with their experience with mentally engaging in future situations and anticipating consequences of behaviors, while young children lack this experience and need better developed EFT abilities to benefit from explicit instructions. Consistent with previous studies reporting positive effects of future thinking on PM in (young) adults [38,40,65], EFT may represent a simple strategy to enhance PM in various age groups, once the ability to mentally engage in future situations is sufficiently developed. Conceptually, our findings provide first evidence for the importance of ‘elaborate’ intention encoding for later intention initiation/execution in children. Pre-experiencing the prospective situation during intention encoding seems to reduce PM task demands, which in turn improves children’s abilities to initiate future intentions (albeit only slightly in younger children). Whether those beneficial effects are due to a stronger cue-action association or reduced retrospective memory demands following deeper task encoding remains an open issue. Altgassen, Rendell [25] reported no differential effects of EFT instructions on event- and time-based PM and argued that EFT may lead to deeper task encoding rather than a stronger cue-action relation because with time-based tasks no explicit cue is provided (in contrast to event-based PM tasks, where external cues can be included when imagine the future situation). Although Altgassen, Rendell [25] did not directly assess retrospective memory, they found better plan adherence following EFT instructions, which may point to deeper task encoding rather than a stronger cue-action link. Consistently, Altgassen, Kretschmer [48] found adolescents to improve their PM performance following the instruction of future thinking, but also when participants were repeatedly exposed with the PM cues (repeated-encoding condition). The authors concluded that stronger memory traces might be the driving mechanism underlying the effects of future thinking in PM. Altgassen, Kretschmer [48] reported improved retrospective memory for the PM cues in the future thinking and the repeated-encoding condition as compared to a standard encoding condition. In the present study retrospective memory for the PM target cues was not assessed which limits the interpretation of the EFT effects. To get a better understanding of the mechanisms that drive the beneficial effects of EFT on children’s PM future studies should test whether the beneficial effects that were found in the present study follow deeper PM task encoding or the formation of a strong cue-action link. Therefore, in addition to PM performance, retrospective memory for the PM cues should be included. Another important issue that should be in the focus of future research is the age from which children can use EFT as an effective strategy to improve PM. Together, our results support the importance of intention encoding for PM suggested by the multiprocess framework [22]. This is the first study to demonstrate this in children. However, results have to be interpreted with caution given that the conclusions about the differential impact of EFT encoding on younger and older children’s PM were drawn on the basis of planned post-hoc comparisons, while the interaction between age group and encoding condition itself was not significant. Moreover, even though we tried to adapt ongoing task difficulty to both age groups, significant group differences in ongoing task performance were found. Specifically, compared to the baseline ongoing task condition the younger age group deteriorated more strongly in the dual-task condition. This is in line with Mahy, Moses’ [11] suggestion that individuals with less cognitive resources (in this study preschoolers) are more strongly affected by adding another task to the ongoing task (see also [55] for similar effects). However, importantly, controlling for baseline ongoing task performance only influenced the age group effect, while the effect of EFT encoding was still significant.

Addressing the second research question of the influence of self-projection on the effects of EFT instructions on PM performance, correlational results indicate a possible influence of self-projection on the effects of EFT encoding on PM performance (i.e., significant relations between PM and self-projection only in the EFT but not the standard encoding condition). However, this trend was not reflected in results of the ANCOVA. The effects of EFT encoding on PM did not change when controlling for self-projection. In contrast, self-projection had an independent significant impact on PM performance and thus seems to be an important mechanism that influences the performance of delayed intentions. The still significant effect of EFT instructions on PM performance after controlling for self-projection (as indicated by ToM) provides the first evidence that, in children, EFT effects on PM performance might result from deeper encoding following the repeated exposure with the PM task rather than from projecting yourself into the future and pre-experiencing the future situation. If self-projection would have been the core mechanism, effects of EFT encoding on PM performance should have been no longer significant, when controlling for self-projection. However, one possible aspect limiting these conclusions might be that ToM tasks were applied as measures of self-projection and may not be suitable to adequately represent self-projection as it is needed in EFT. Consistently, Hanson, Atance [53] found no significant relation between EFT and ToM and argued that changing one’s own perspective into a future perspective may differ from changing one’s own perspective to that of another person. Thus, self-projection as measured by ToM tasks, may cover different aspects of self-projection than those needed for projecting the self into the future. However, in contrast neuroscientific observations argue against this claim and for a connection between EFT and ToM [50]. For instance, Fair, Cohen [66] reported that the connectivity of the so called ‘default mode network’ (a brain network where EFT and ToM are supposed to be located) is still developing throughout childhood and only appears to form an interconnected network later in life. Thus, effects of EFT instructions on PM performance might be influenced by ToM (resp. self-projection) once underlying brain structures form an integrated network. This needs to be addressed by future studies targeting different age groups across childhood and applying MRI measures in addition to behavioral assessments to investigate whether the strength of connections within the ‘default mode network’ affects relations between EFT and ToM as well as their contributions to PM.

Another interesting result found in the present study was that the age group effect on PM performance was no longer significant when including self-projection as a covariate. Previous studies mainly focused on executive function improvements when explaining PM age differences in children and adolescence (see [11] for a model explicitly addressing this issue). Results of the present study showed that in addition to executive functions the ability for self-projection may be particularly important when considering PM age effects in younger age groups. Consistently, Ford, Driscoll [19] reported a robust contribution of ToM to PM in younger children using a correlational design. Thus future studies should not only vary executive control demands when investigating PM age effects, but also the necessity of mental self-projection to investigate its impact on the PM success.

An aspect that may limit the conclusions drawn from the present results is that the influence of self-projection on the effects of encoding condition (and age) was investigated using an exploratory ANCOVA. Applying this analysis to data sets that do not fully meet all requirements of an ANCOVA has been contrarily discussed in previous publications (see e.g. [67,68]). The statistical requirements for conducting an ANCOVA (specifically, nonrandom group assignment and no group differences on the covariate, [68]) were only partly fulfilled. Specifically, both age groups were predefined and children could not be randomly assigned to one of the two age groups. Further, both age groups differed significantly with regards to the covariate (school-aged children > preschoolers, *p* < .001). Thus it cannot be fully excluded that the covariate removes considerable variance of the independent variable [68]. Here, results need to be interpreted with caution and conclusions can only be seen as preliminary. However, despite nonrandom group assignment (i.e., preschoolers and school-aged children) as in the present study, ANCOVAs can be considered as being useful to explore and better understand the pattern of shared variance in a given dataset (see e.g. [69] for a study using a similar approach). Importantly, individuals within both age groups were randomly assigned to one of both encoding conditions. Moreover, both encoding groups did not differ significantly with regards to the covariate, neither within the preschool (*p* = .93) nor within the school-aged group (*p* = .08). Thus, for a first exploration of the impact of self-projection on the effects of EFT encoding on PM, the ANCOVA approach seems suitable.

Although the present research provides new insights into the concept of future thinking (especially in children) and its relation to PM and self-projection, a more detailed understanding of future thinking is required to obtain a clearer understanding of its underlying mechanisms. Here, executive functions might be prominent candidates. Our results show that a population with developing executive functions benefits from EFT. However, it is unclear whether EFT instructions are more effective when executive functions are further developed given that 5-year-olds’ PM did not improve significantly. Studies on clinical populations that indicate relations between future thinking and cognitive control (see e.g. [70] for a study on Parkinson’s disease) suggest a role for developing executive functions. Future studies should include independent executive function measures and test how much variance of future thinking abilities can be explained by these measures.

In addition to existing evidence for positive effects of EFT instructions on older adults’ [25] and adolescents’ [48] PM, our study is the first to extend these findings to younger age groups with still developing cognitive control functions. Specifically, we provided first evidence for beneficial effects of EFT encoding on PM in school-aged children, which on the one hand indicated that already children could make use of EFT as an encoding strategy. On the other hand, results also showed that future thinking abilities (and possibly executive functions) might have to be sufficiently developed to facilitate PM performance, as preschool children’s PM was not significantly improved when instructing EFT. Moreover, our results showed that the development of self-projection as measured by ToM could not explain the positive effects of EFT encoding on PM and thus call for further research disentangling the specific mechanism(s) that may underlie EFT effects.

## Supporting Information

S1 Dataset This dataset includes all collected data that were analyzed for the present study. (SAV)
